# Adaptive response of *Rhodococcus opacus* PWD4 to salt and phenolic stress on the level of mycolic acids

**DOI:** 10.1186/s13568-016-0241-9

**Published:** 2016-09-08

**Authors:** Carla C. C. R. de Carvalho, Martin A. Fischer, Sandra Kirsten, Birgit Würz, Lukas Y. Wick, Hermann J. Heipieper

**Affiliations:** 1 iBB-Institute for Bioengineering and Biosciences, Instituto Superior Técnico, Universidade de Lisboa, Av. Rovisco Pais, 1049-001 Lisbon, Portugal; 2 Department of Environmental Biotechnology, Helmholtz Centre for Environmental Research-UFZ, Permoserstr. 15, 04318 Leipzig, Germany; 3 Department of Environmental Microbiology, Helmholtz Centre for Environmental Research-UFZ, Permoserstr. 15, 04318 Leipzig, Germany

**Keywords:** Mycolic acids, Adaptation, *Rhodococcus opacus*, Salt stress, Solvents, Membrane fatty acids

## Abstract

Mycolata form a group of Gram-positive bacteria with unique cell envelope structures that are known for their high tolerance against antibiotics and both aromatic and aliphatic hydrocarbons. An important part of the unique surface structure of the mycolata is the presence of long chain *α*-alkyl-β-hydroxy fatty acids, the mycolic acids. In order to investigate the adaptive changes in the mycolic acid composition, we investigated the composition of mycolic acids during the response both to osmotic stress caused by NaCl and to 4-chlorophenol in *Rhodococcus opacus* PWD4. This bacterium was chosen as it is known to adapt to different kinds of stresses. In addition, it is a potential biocatalyst in bioremediation as well as for biotechnological applications. In the present study, cells of *R. opacus* PWD4, grown in liquid cultures, responded to toxic concentrations of NaCl by increasing the ratio between mycolic acids and membrane phospholipid fatty acids (MA/PLFA-ratio). Cells reacted to both NaCl and 4-chlorophenol by decreasing both the average chain length and the unsaturation index of their mycolic acids. These changes in mycolic acid composition correlated with increases in cell surface hydrophobicity and saturation of membrane fatty acids, demonstrating the relation between mycolic acid and phospholipid synthesis and their contribution to cell surface properties of *R. opacus* PWD4.

## Introduction

The *Rhodococcus* cell envelope is characterized by the presence of long chain α-alkyl-β-hydroxy fatty acids, the mycolic acids (MA) (Asselineau and Lederer 1950). These mycolic acids, which are linked to an arabinogalactan complex or attached to a trehalose disaccharide, form an outer lipid layer. This layer is characteristic for the taxon mycolata and is found in eight other genera: *Corynebacteria, Dietzia, Gordonia, Mycobacteria, Nocardia, Rhodococcus, Skermania* and *Tsukamurella* (Sutcliffe 1998). The presence of MA is one of the reasons for the high tolerance of these bacteria to antibiotics and other forms of environmental stress (Alvarez et al. 2004; Barkan et al. 2009). The length, structure and complexity of the MA vary between the different genera and also between different strains, which can be used as a marker for classification (Kellogg et al. 2001). MA in *Rhodococcus* have an average chain length of 28–54 carbon atoms and up to four double bonds can be found in the their distal region. In this genus, the alkyl side branch usually consists of a carbon chain with 10–16 carbon atoms and no functional groups. The longer meromycolate chain with 20–42 carbon atoms can contain up to four double bonds (Stratton et al. 2003).

The synthesis of MA is based on a unique long chain fatty acid synthesizing enzymatic cascade. Unlike other bacteria, the fatty acid synthase system (FAS) of *Rhodococcus* and other genera in the mycolata consists of two FAS systems (Sutcliffe 1998). For de novo synthesis of fatty acids, a synthase system quite similar to the mammalian like FAS-I system is used. The products of this first FAS-I system are the fatty acids and also the educts for the synthesis of the mycolic acids. The FAS-II system of the mycolata is more similar to the fatty acid synthase system found in bacteria and consists of various dissociable subunits fulfilling the necessary reactions for chain elongation of the meromycolate chain (Asselineau and Lederer 1950; Barry et al. 1998; Nishiuchi et al. 2000; Takayama et al. 2005).

Changes in the MA profile as a response to the presence of different aromatic compounds or to the hydrocarbons used as carbon and energy source have been shown (Sokolovska et al. 2003; Wick et al. 2002). Several members of the mycolata have been investigated concerning the changes of their mycolic acid patters under various conditions. Wick et al. (2003) observed for *Mycobacterium frederiksbergense* LB501T correlations between growth on anthracene and glucose as substrates and changes in the mycolic acid profile of the bacterium. It was observed that, according to the mixing ratio between the two substrates, different clusters of mycolic acids were present in the cell envelope. In addition to different substrates, also variations in the cultivation techniques, both in suspensions or in form of biofilms, were investigated and showed that these alterations were also influencing the mycolic acid composition (Wick et al. 2003).

The influence of different carbon sources on the composition of the mycolic acids of *Rhodococcus erythropolis* E1 was investigated by Sokolovská et al. (2003). Analysis showed that, cells grown on compounds with even-numbered carbon chains synthesized almost only even-numbered mycolic acids, whereas during growth on odd-numbered carbon chained substrates both even- and odd-numbered mycolic acids were found. Moreover, the use of humic acids and phenols as a carbon and energy sources resulted in an increase in the degree of saturation in different *R. erythropolis* strains (Kolouchova et al. 2012). In addition to these observations, Stratton et al. (2003) observed changes in the different growth phases of cultures and described a decrease of saturated mycolic acids with an increasing culture age. These adaptive mechanisms, in combination with the great variety of metabolic pathways and abilities of the genus *Rhodococcus*, are responsible for the interest and relevance of these bacteria in terms of biotransformation and bioremediation.

The ability of *Rhodococcus* and other members of the mycolata to cope with organic substrates like phenol and BTEX compounds as well as to the toxicity of high concentrations of compounds such as chlorophenols and terpenes have been observed (de Carvalho et al. 2004, 2009; Tsitko et al. 1999). Recently, de Carvalho and colleagues (de Carvalho et al. 2014) described the high tolerance and adaptive potential of a *Rhodococcus* strain under salt stress. The high tolerance of *Rhodococcus* strains is correlated with their unique cell envelope (Alvarez et al. 2004; Barkan et al. 2009; de Carvalho 2012). The objective of this study was thus to evaluate the adaptive changes occurring at the mycolic acid level in *R. opacus* PWD4 under different conditions. In both natural and man-made environments, *Rhodococcus* cells may be exposed to extreme or fluctuating water activities/osmotic stress due to e.g. drought stress or cyclic desiccation and rehydration events. On the other hand, strains of *Rhodococcus* are ubiquitous and should be exposed to compounds such as chlorophenols which are easily found in industrial waste, pesticides and insecticides, being recalcitrant to degradation (Jensen 1996). However, little is known about the actual changes occurring at the level of the mycolic acid composition when *Rhodococcus* cells are exposed to osmotic stress and organic compounds not used as carbon sources. If the adaptive response to both stresses is similar is also unknown.

For this investigation, an up and running protocol for mycolic acid extraction and analysis was first established. With this new tool, changes in the mycolic acid composition in the presence of osmotic stress (NaCl) and 4-chlorophenol, as standard compound for toxic aromatic compounds, were assessed.

MA biosynthesis is closely related to that of membrane fatty acid. They are located on the outer surface of the bacterial envelope and might have an effect on surface properties. Therefore, the obtained results were correlated with already established physiological parameters, namely the fatty acid composition and the cell surface hydrophobicity determined by the water contact angle.

## Materials and methods

### Culture conditions and chemicals

*Rhodococcus opacus* PWD4 (DSM 44313) (Duetz et al. 2001) was cultivated in a mineral medium as described by Hartmans et al. (1989), with 4 g/L disodium succinate as carbon and energy source. Cells were grown in 50 mL liquid cultures at 30 °C in a horizontally shaking water bath at 180 rpm. All chemicals were reagent grade and obtained from commercial sources.

The stressors were added to the cultures in the early exponential growth phase (OD_560nm_ between 0.4 and 0.6). Cells were harvested 2 h after stressor application for fatty acid analysis and determination of water contact angle. The cell suspensions were centrifuged at 10,000*g* for 10 min at 4 °C. The cell pellets were frozen for fatty acid analysis or stored at 4 °C for water contact angle measurements.

Toxicity of the stressors added to cells growing in the early exponential phase was estimated by the effective concentration 50 % (EC50), i.e. the concentration that causes a 50 % inhibition of bacterial growth as described earlier by Heipieper et al. (1995). Growth inhibition caused by the toxic compounds was measured by comparing the differences in growth rate μ (h^−1^) between intoxicated cultures (µ_stressor_) with that of control cultures (µ_control_). The growth inhibition of different concentrations of stressors was defined as the percentage of the growth rates of intoxicated cultures and that of control cultures without stressor addition.

### Mycolic acid preparation

For the mycolic acid analysis we used a protocol modified from Sokolovská et al. (2003). Therefore, 30–50 mg of cells harvested in the late exponential growth phase were resuspended in 2 mL of minimal media. An equal volume of 25 % KOH (w/w) in 50 % ethanol–water (v/v) was added to the suspension. The mixture was then incubated for 1 h at 120 °C. Afterwards, 1.75 mL of concentrated HCl were added to the solution and the fatty acids and the mycolic acids were extracted with 3 mL dichloromethane for 3 min on a Vortexer at room temperature. This extraction procedure was done three times. The organic phases were combined and evaporated under nitrogen flux until a volume of approximately 1 mL and then transferred to a GC-vial and therein evaporated to dryness. 100 µL of BSTFA derivatization reagent and 50 µL of pyridine were added to the dried fatty acids and softly shaken. The mixture was incubated at 80 °C for 20 min. After the incubation, the mixture was evaporated under nitrogen flux. To purify the reaction product, 1 mL of benzene was added to the dried sample and evaporated under nitrogen flux. This purification step was repeated once. The purified, dried sample was taken up in 100 µL hexane and transferred into a GC-vial with micro insert. The sample was stored until analysis at −20 °C.

### Gas chromatographic analysis of mycolic acids

Mycolic acids were analyzed on an Agilent 5975T GC–MS System (AgilentTechnologies, Santa Clara, CA, USA). The column used was a Zebron ZB-1MS capillary column (Phenomenex Inc, Torrance, CA, USA; length, 12 m; inner diameter, 0.20 mm; 0.33 µm film thickness), 100 % Dimethylpolysiloxane, 30 m in length, with an internal diameter of 0.18 mm and 0.18 µm of film thickness. Helium was used as mobile phase and 1 µL of sample was injected for analysis in splitless mode, with injector temperature set at 140 °C. The oven temperature program consisted of an initial steady temperature plateau at 50 °C for 1 min, followed by two consecutive ramps at 10 °C/min from 50 to 120 °C, and at 3 °C/min from 120 to 360 °C. Post run column regeneration was performed at 360 °C for 5 min.

### Lipid extraction, transesterification, and fatty acid analysis

The lipids were extracted with chloroform/methanol/water as described by Bligh and Dyer (1959). Fatty acid methyl esters (FAME) were prepared by incubation for 15 min at 95 °C with boron trifluoride/methanol according to the method of Morrison and Smith (1964). FAME were extracted with hexane and analysed by GC-FID.

### Analysis of fatty acid composition by GC-FID

Analysis of FAME in hexane was performed using a HP5890 GC System (Hewlett & Packard, Palo Alto, USA) equipped with a split/splitless injector and a flame ionisation detector (FID). A CP-Sil 88 capillary column (Chrompack, Middelburg, The Netherlands; length, 50 m; inner diameter, 0.25 mm; 0.25 µm film thickness) was used for the separation of the FAME. GC conditions were the following: injector temperature was held at 240 °C, detector temperature was set at 270 °C. The injection was made in splitless mode, and the carrier gas was He at a flow of 2 mL min^−1^. The oven temperature program was as follows: 40 °C, 2 min isothermal; 8 °C/min to 220 °C; 15 min isothermal at 220 °C. The peak areas of the FAMEs were used to determine their relative amounts. The fatty acids were identified by GC and co-injection of authentic reference compounds obtained from Supelco (Bellefonte, USA). The degree of saturation of fatty acids was defined as the ratio between the saturated fatty acids (C16:0, C17:0, C18:0) and unsaturated fatty acids (C16:1Δ9*cis*, C18:1Δ9*cis*) present in this bacterium.

### Characterization of bacterial cell surface hydrophobicity

Physico-chemical cell surface properties of bacterial cells were investigated using water contact angle measurements as described by others (Van Loosdrecht et al. 1987). Bacterial lawns needed for contact angle (θ_w_) measurements were prepared by collecting cell suspensions in 10 mM KNO_3_ on 0.45-μm pore-size Micropore filters (Schleicher & Schuell, Dassel, Germany), mounting the filters on glass slides, and drying them for 2 h at room temperature. Cell surface hydrophobicities were derived from θ_w_ of water drops on the bacterial lawns using a Krüss drop shape analysis system DSA 100 (Krüss GmbH, Hamburg, Germany). According to an earlier classification, cells exhibiting contact angles of θw < 20°, 20° ≤ θw ≤ 50° and θw > 50° were considered hydrophilic, intermediately hydrophilic and hydrophobic, respectively (Van Loosdrecht et al. 1987).

### Statistics

All experiments were carried out in triplicate. All figures show mean values with the corresponding standard deviations as error bars.

## Results

### Effect of NaCl on growth and membrane fatty acid composition

**Fig. 1:**
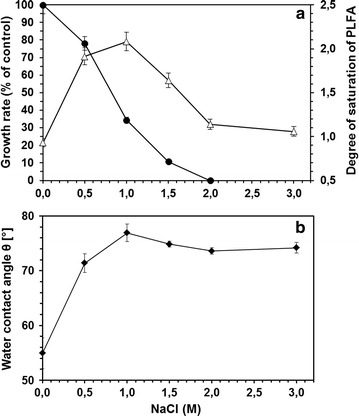
Effect of NaCl on (**a**) growth (*filled circle*), degree of saturation of phospholipid fatty acids (*triangle*) and (**b**) water contact angle (*filled diamond*)

Cells of *R. opacus* were cultivated in mineral medium and NaCl was added in different concentrations during the early exponential growth phase. A clear concentration dependent growth inhibition pattern of the *R. opacus* population could be observed (Fig. 1a). A complete inhibition of growth was observed at NaCl concentrations of 2 M with an effective concentration reducing population growth by 50 % (EC50) of about 0.9 M, revealing that *R. opacus* is a halotolerant microorganism. The bacterial cells responded to increasing concentrations of NaCl by increasing both the degree of saturation of the membrane phospholipid fatty acids (Fig. 1a) and their cell surface hydrophobicity (Fig. 1b). These two well know adaptive mechanisms showed a maximum in the presence of 1 M NaCl. Curiously, while the cell surface hydrophobicity was maintained around 74 % for NaCl concentration higher than 1 M, the degree of saturation decreased with increasing concentration of NaCl from 1 to 2 M NaCl.

### Effect of NaCl on mycolic acid composition

**Fig. 2:**
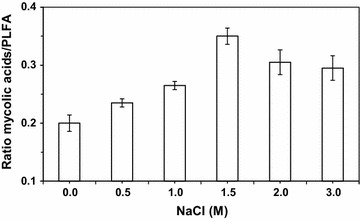
Effect of NaCl on the ratio of total MA and PLFA

The method for extraction and detection of mycolic acids (MA) used in this work also allowed the analysis of phospholipid fatty acids (PLFA). To avoid modifications in the composition of MA caused by different growth phases (Stratton et al. 2003) it was guaranteed that all samplings were carried out during the exponential growth phase. Therefore, we calculated the ratio between the overall abundance of MA and those of PLFA. Interestingly, the overall ratio between MA and PLFA increased with increasing NaCl concentration, whereby a maximum was detected in the presence of 1.5 M NaCl (Fig. 2). Thus, the bacterium nearly doubled the relative amount of MA under stress conditions.

**Fig. 3:**
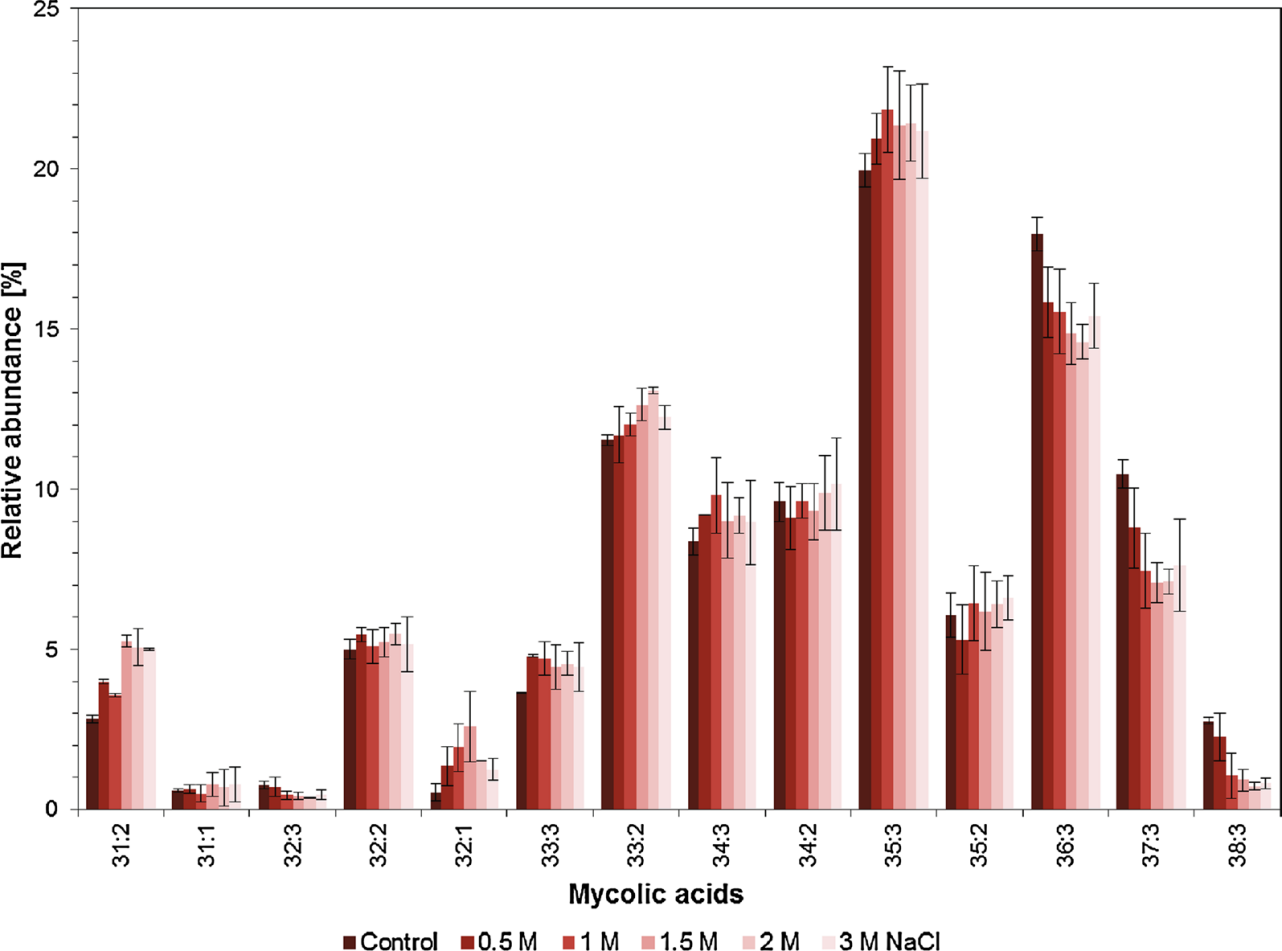
Effect of NaCl on the mycolic acid patterns

**Fig. 4:**
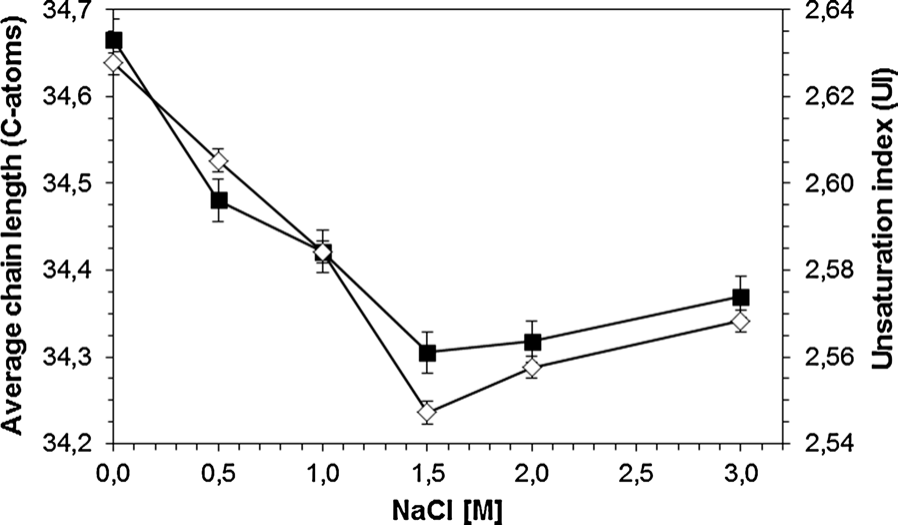
Effect of NaCl on the average chain length (*filled square*) and unsaturation index (*diamond*) of mycolic acids

The qualitative analysis of MA of cells grown in the absence of NaCl showed a typical profile of chain length between C31 and C38 of the MA (Fig. 3). No saturated MA could be detected, and the unsaturation varied between monounsaturated and triple unsaturated MA with 35:3 being the predominant MA. In the presence of NaCl, the MA profile showed significant modifications when compared to the control cells. These changes can be expressed as the average chain length (ACL) of the MA as well as the unsaturation index (UI) which is the average amount of double bonds present in fatty acid chains (Kaszycki et al. 2013). Thus, the higher the UI is, the more fluid the cell wall becomes due to a lower melting temperature of the corresponding MA. In the present study, both ACL and UI values decreased with increasing salt concentration up to 1.5 M NaCl but an increase was observed for higher salt concentrations (Fig. 4).

### Effect of 4-chlorophenol on cell growth and mycolic acid composition

In order to test whether the observed adaptive responses of *R. opacus* PWD4 cells were limited to osmotic stress or whether they may express a more general stress response mechanism, we carried out similar experiments with 4-chlorophenol (4CP). This compound is one of the standard monoaromatic chemicals used to investigate cellular toxicity and adaptation (Chrzanowski et al. 2009).

**Fig. 5:**
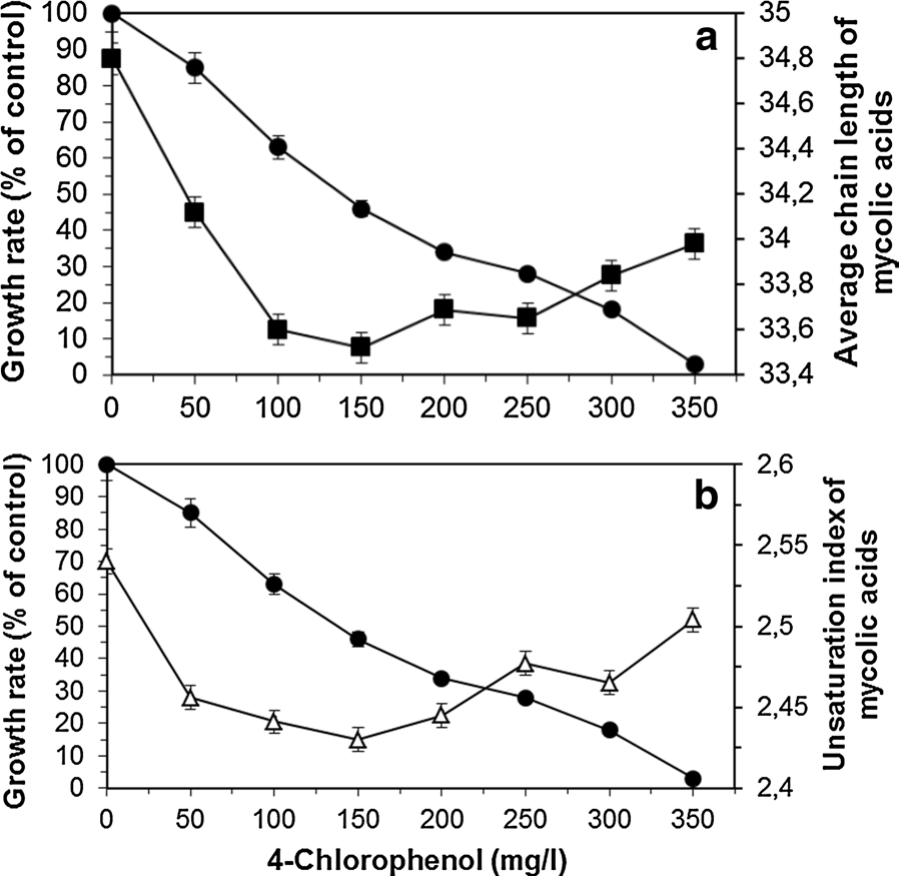
Effect of 4-chlorophenol on (**a**) growth (*filled circle*), and on the average chain length (*filled square*) and (**b**) unsaturation index (*triangle*) of mycolic acids

A complete inhibition of growth was observed at 4CP concentrations of 350 mg/L, with an effective concentration reducing growth by 50 % (EC50) at about 150 mg/L (Fig. 5a). Besides, in the presence of 4CP, the cells showed a concentration dependent modification of their MA content. Both the ACL (Fig. 5a) and UI (Fig. 5b) of the MA decreased with increasing concentration of 4CP up to 150 mg/L. For higher 4CP concentrations, both ACL and UI increased.

## Discussion

*Rhodococcus* is a mycolic acid-containing genus, with MA constituting the most characteristic component of the cell wall and serving as chemotaxonomic index. The MA can be represented by the degree of unsaturation, the average carbon number and the structure of their α-alkyl branch. *Rhodococcus* species such as *R. equi* and *R. erythropolis* belong to the short MA chain-length group with an average carbon number in the 30 s and presenting saturated fatty acids, whilst species such as *R. fascians* and *R. luteus* present on average MA with 40 carbons containing up to 4 double bonds (Nishiuchi et al. 2000).

In the present study, *R. opacus* PWD4 contained mainly MA with 31–38 carbon atoms with up to 3 unsaturations, with the MA 35:3 being the most abundant (Fig. 3). Curiously, the cells produced MA with considerable amounts of both odd- and even-numbered carbon chains, contrarily to what is usually observed in *Rhodococcus* species (Nishiuchi et al. 2000). Sokolovská et al. observed that *R. erythropolis* E1 lacked odd-numbered carbon chains when the cells grew on linear alkanes with even number of carbons but the cells produced MA with both even- and odd-numbered carbon chains if the carbon source was branched alkanes or mixtures of compounds (Sokolovska et al. 2003). In the present study, the succinate used as carbon source resulted in the production of both types of MA. Besides, the presence of NaCl did not alter the composition of carbon chain length of the MA, only the relative amount of each type of MA (Fig. 3).

The *R. opacus* PWD4 cells increased the ratio of MA over PLFA as response to the presence of salt in the growth media, with a maximum being achieved at 1.5 M NaCl (Fig. 1). Similarly, curves showing a distinct maximum response were achieved for the degree of saturation of PLFA (with a maximum at 1 M NaCl; Fig. 1) and for the unsaturation index of MA (minimum at 1.5 M NaCl; Fig. 1). At these concentrations, the cells presented the highest contact angles (Fig. 1) but presented considerable low growth rates (Fig. 1), which could be an indication of the metabolic burden associated to the modifications in the MA and PLFA compositions to survive in the presence of the salt. Similar responses had been observed with *R. erythropolis* DCL14 exposed to NaCl (de Carvalho et al. 2014), and to extreme values of temperature and pH (de Carvalho 2012). Stratton et al. (2003) also observed different MA profiles in three *Rhodococcus* isolates with culture age and growth temperature for cells grown on Tween 80 or glucose (Stratton et al. 2003). In fact, the latter study is an example of the diversity of changes in MA composition that may be observed as response to environmental changes.

The average chain length of MA decreased in the presence of both NaCl and 4CP up to a concentration of 1 M NaCl and 150 mg/L 4CP, respectively. This is in accordance with known adaptive responses of bacteria to solvents or increase in temperature, where an increase in the degree of saturation (decrease in UI) is always related to a decrease in the chain length (Heipieper and de Bont 1994; Rock 1984). Thus, the results of our studies clearly suggest that an active response mechanism, similar to that known for the adaptation of PLFA fatty acids to several kinds of stresses (Alvarez et al. 2004), is also reflected in the adaptation of MA content of *R. opacus*. In *Nocardia asteroides*, an increased number of polyunsaturated MA was observed at low temperatures whilst more saturated MA were produced at higher temperatures, but no significant differences were observed in the carbon chain length of the MA (Tomiyasu 1982). However, studies with *M. phlei* showed that this bacterium regulates cell wall fluidity by decreasing the MA chain length at low temperatures (Liu et al. 1996) whilst the inability of *M. tuberculosis* to regulate the fluidity of the cell wall and to synthesize MA at low temperature resulted in loss of viability (Takayama et al. 1978). *Rhodococcus opacus* cells increased the surface hydrophobicity with increasing concentrations of NaCl, reaching a contact angle plateau at ca. 75º (Fig. 1). Bendinger and co-workers related the chain length of MA in coryneform bacteria with their physicochemical cell surface and adhesive properties (Bendinger et al. 1993). *Rhodococcus erythropolis*, *R. rhodochrous* and *R. globerulus* with MA with 34–48 carbons presented contact angles between ca. 70°–100° (in accordance to the value attained in the present study), which is considered an hydrophobic character.

We can thus state that *R. opacus* PWD4 cells respond to osmotic and solvent stress by changing their MA content, decreasing both their unsaturation index and average chain length. This is very similar to modifications observed in the membrane PLFA contents of these bacteria as a response to stresses. These mechanisms lead to less fluid membranes, more hydrophobic MA and increased cell surface hydrophobicity. These concentration dependent changes in the mycolic acid composition as a reaction to different substrates had already been previously reported (Wick et al. 2003; Sokolovska et al. 2003) and correlated with other physiological parameters such as the fatty acid profile and the zeta potential. So far, only very little is known of the role that mycolic acid may have under osmotic stress conditions (Alvarez et al. 2004). However, our study suggest that the ability of *R. opacus* cells to adapt their MA profile, by decreasing both their unsaturation index and average chain length, which leads to less fluid membranes and increased cell surface hydrophobicity, may be used to increase the bioremediation potential of this bacterium to degrade hydrophobic compounds in saline environments.

## Abbreviations

4CP: 4-chlorophenol
EC50: effective concentration 50 %
FAME: fatty acid methyl esters
FAS: fatty acid synthase system
FID: flame ionisation detector
MA: mycolic acids
PLFA: phospholipid fatty acids
UI: unsaturation index
